# Improved Dynamic Obstacle Mapping (iDOMap)

**DOI:** 10.3390/s20195520

**Published:** 2020-09-26

**Authors:** Ángel Llamazares, Eduardo Molinos, Manuel Ocaña, Vladimir Ivan

**Affiliations:** 1 Department of Electronics, University of Alcalá, 28801 Madrid, Spain; mocana@depeca.uah.es; 2 Karlsruhe Institute of Technology, 76131 Karlsruhe, Germany; eduardo.molinos@kit.edu; 3 Institute of Perception, Action and Behavior, University of Edinburgh, Edinburgh EH8 9YL, UK; v.ivan@ed.ac.uk

**Keywords:** Dynamic Obstacles Mapping (DOMap), Particle Filter, optical flow, dynamic occlusion detector

## Abstract

The goal of this paper is to improve our previous Dynamic Obstacle Mapping (DOMap) system by means of improving the perception stage. The new system must deal with robots and people as dynamic obstacles using LIght Detection And Range (LIDAR) sensor in order to collect the surrounding information. Although robot movement can be easily tracked by an Extended Kalman Filter (EKF), people’s movement is more unpredictable and it might not be correctly linearized by an EKF. Therefore, to deal with a better estimation of both types of dynamic objects in the local map it is recommended to improve our previous work. The DOMap has been extended in three key points: first the LIDAR reflectivity remission is used to make more robust the matching in the optical flow of the detection stage, secondly static and a dynamic occlusion detectors have been proposed, and finally a tracking stage based on Particle Filter (PF) has been used to deal with robots and people as dynamic obstacles. Therefore, our new improved-DOMap (iDOMap) provides maps with information about occupancy and velocities of the surrounding dynamic obstacles (robots, people, etc.) in a more robust way and they are available to improve the following planning stage.

## 1. Introduction

Autonomous navigation systems have been studied extensively in the literature [1,2]. Such systems should be able to perform perception, localization, planning and actuation. To perceive the state of the environment, on-board sensors are used. The localization stage is usually based on a fusion of sensory information and a priori map to determine the location of the robot within the global frame. Such maps are usually obtained in a semi-autonomous way as proposed by [3,4,5] or using Simultaneous Localization And Mapping (SLAM) techniques [6] to reduce the uncertainty of localization and mapping processes by doing both at the same time [3,7]. Once the robot location has been determined, the sequence of actions necessary to reach the goal can be computed within the planning stage, which can be divided in global and local. The global resulting plan is usually a mesh of connected waypoints that must satisfy various constraints such as: holonomic constraints, traffic rules, energy efficiency, safety, etc. To take care of the safety, in the most of the cases a local planner must deal with the obstacle avoidance [8,9,10].

To perform the local or global path planning, a good representation of the environment or map is needed. It can be local to represent the surroundings of the robot (most suitable for local navigation) or global to represent the whole environment (in order to execute path planning tasks). The problem of mapping is based on acquiring a spatial model of environment that surrounds the robot. These models can be classified in metric or topological approaches. In the first group, geometric properties of the environment are taken into account, like the occupancy grid-mapping proposed in [11] or using polyhedric approach like in [12]. The second group, a set of nodes at relevant places are connected by means of arcs that store information that helps to the global planner [13].

Although these mapping methods can be useful for the most of the obstacle-avoidance algorithms in static or quasi-static environments, when they have to deal with a high number of dynamic objects it might not be enough and some local planners have to include new innovations [14,15]. It is because they only take into account the occupancy of static obstacles providing, therefore, an incorrect modelling of the dynamic ones. For that reason, it is recommended that the map includes new predicting information than can help following planning stages. One implementation is Grid-Based DATMO (Detection And Tracking of Moving Obstacles) [16] mapping methods.

Grid-Based DATMO methods represent the environment as an occupancy grid, and they include information about dynamic objects by means of tracking each cell instead of segment the environment into objects to track them. These approaches avoid the object concept, also the problem of multi-object detection and tracking, which is sometimes very difficult to solve. For that reason, the advantage of these methods is that the computational cost is proportional to the grid size and the number of cells, but not the number of obstacles. Other advantage of these methods is that the sensor data fusion from different sensors can be computed at the raw data level using occupancy grid maps, where the data association is not needed. One of these approaches is based on the Evidence Theory, also called Dempster-Shafer theory [17] taking into account the inconsistencies between consecutive grids as evidence of conflict. This method has the advantage of modelling a cell in three states: free, occupied or not observed yet.

Although some works by [18,19,20] are based on these methods, due to the amount of parameters tuning needed, it is usually a challenging task. Another popular approach is the Bayesian Occupancy Filter (BOF) [21,22].

The BOF evaluates the environment occupancy regardless of the kind of the object. The occupancy of the cells is computed as an probabilistic formulation initially proposed by [11], dealing with uncertainty due to the noise or the inaccurate sensors through the Bayes theorem. The BOF is described as a representation of the space in a cell grid. Each cell describes a portion of the environment and includes different kinds of information about this part of the space, such as occupancy, velocities, riskiness, etc.

Based on these characteristics and in order to deal with different participants as dynamic obstacles without needed to use several models for each of them, we proposed a probabilistic model of the environment in a previous work [23], using a Grid-Based DATMO with BOF filter approach. This system was called Dynamic Obstacle Map (DOMap).

In this paper, we present an improvement of our previous DOMap, a Grid-Based DATMO, by means of three key points: first introducing the laser reflectivity in the movement detection stage, secondly proposing a method to handle the occlusions and finally using a Particle Filter (PF) instead of Extended Kalman Filter (EKF) to deal with dynamic obstacles with different motion patterns like people. Paper is organized as follows: first proposal is introduced, then test bed and results will be presented and finally conclusions and future works will be detailed.

## 2. Proposal

Our previous work DOMap [23] was a probabilistic model of the dynamic environment using: (1) an extension of the Bayesian Occupancy Filter proposed by [21] to a three-dimensional method to detect the obstacles positions and (2) a method to estimate the velocity of these obstacles using a tracking stage based on optical flow and a detection stage based on blob filtering.

**Figure 1:**
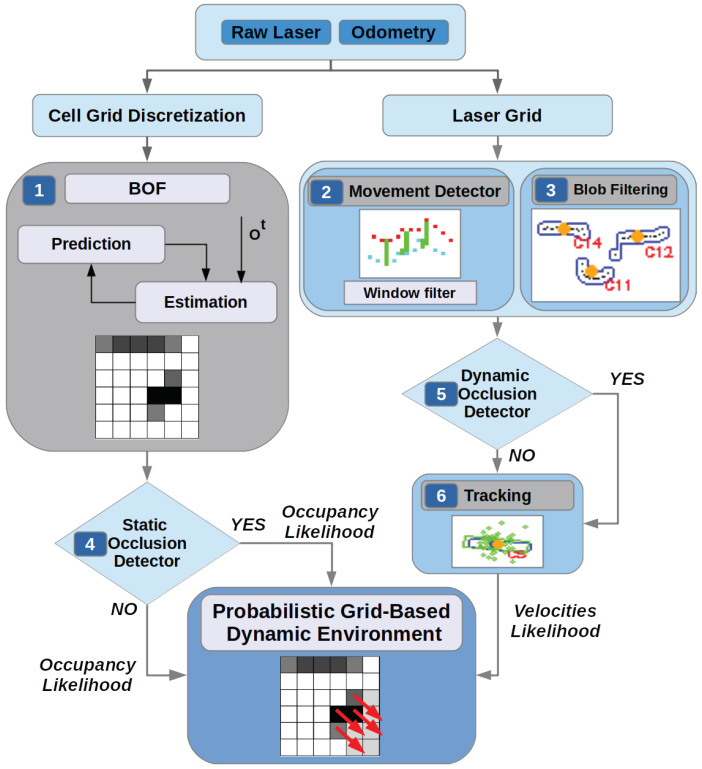
Proposed iDOMap diagram.

In this section, we are going to show the differences respect to our previous work. Figure 1 shows the proposed structure with the following improvements: (1) the Movement Detector (block 2 in the Figure 1) has been improved including the laser reflectivity remission in the optical flow matching stage (Section 2.1.1), (2) the occlusions have been handled in two ways with several blocks added: a Static Occlusion Detector (block 4 in the Figure 1) and a Dynamic Occlusion Detector (block 5 in the Figure 1) (Section 2.2), and finally (3) the Tracking (block 6 in the Figure 1) has been improved using a Particle Filter (PF) instead of an EKF as presented before and it will be explained in Section 2.3. With these improvements, the new proposal is going to take into account participants of unusual appearance, such as people wearing fully loose clothes or pushing objects (such as baby carriage, shopping cart, baggage or trolleys) or even small participants, such as kids or pets. This unlimited number of kinds of participants makes it impossible to model each of them, making necessary a grid-based model-free proposal. We have named this new algorithm improved-DOMap or iDOMap.

### 2.1. Evaluation of Movement Detection

To improve the robustness of the movement detection of our system, the accuracy and richness of the data are crucial. In this section we evaluate the impact of using laser reflectivity in our Movement Detector, by means of improving the data association in the optical flow algorithm.

#### Laser Reflectivity

**Figure 2:**
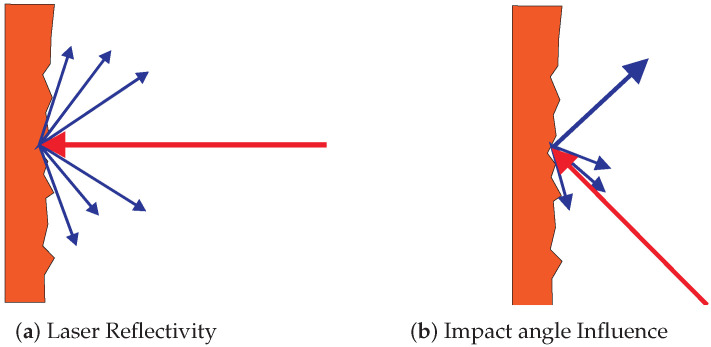
Laser reflectivity in real experiments.

The movement detection in DOMap was based on optical flow. In order to improve the performance of this algorithm, it would be appropriate to take into account more values than the ones that system is tracking. For that reason a possible approach could be to take into account the reflectivity, also called Received Signal Strength Intensity (RSSI) or remission. Figure 2a shows laser reflectivity phenomenon, which can be used as intensity to identify objects based on this parameter. It is strongly dependent on the beam impact angle on the object (as shown in Figure 2b) and this angle is influenced by the roughness of the surface [24]. Therefore, this represents a weakness of this method. In addition, the color of the object affects the measurement [25]. Some authors tried to identify the brightness of the objects based on distance and reflectivity without success [26].

Moreover, the manufacturers usually do not provide further information about this parameter. The little information provided about the parameter is not adjusted, which means you can obtain different values from the same object with two different LIDAR devices. Making it even more tricky to take it into account. Above all these difficulties, we have tested the effect of using this parameter inside matching stage of the optical flow to improve the motion detection. Tests have been performed with some of the most commonly used LIDAR in robotics, such as HOKUYO URG-04LX and SICK LMS151. Several tests have been performed with dynamic obstacles, where the reflectivity variation of impacts over the same object ranges from the 10% up to 28%. In spite of this variation, this parameter helps the optical flow in the impacts matching, adding a feature to each impact point, assuming constant intensity between consecutive time steps. This assumption is more accurate in the case of the SICK LMS 151 due to its higher scanning frequency (50 Hz) compared with the HOKUYO URG-04LX (10 Hz).

**Figure 3:**
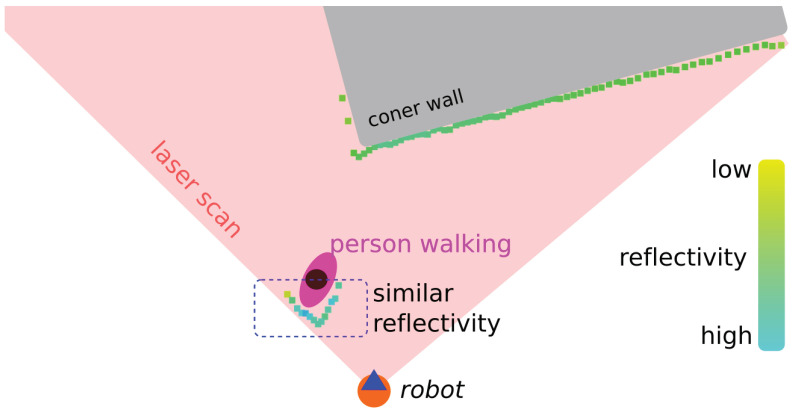
Example of LIDAR reflectivity in real experiment.

Figure 3 shows an example of LIDAR reflectivity in a real experiment, with an orange circle that represents our robot, the blue triangle is the LIDAR, a corner wall in grey color and a person walking (purple oval with a black circle in the middle). Impact dot color represents the reflectivity value that ranges from yellow (low value) to blue (high value). In both objects, the person and the wall can be appreciated how the reflectivity has similar values in the same object.

### 2.2. Occlusion Handling

When the LIDAR is mounted in a robot, the occlusions between objects, from the LIDAR point of view, is a common situation in dynamic environments. This problem appears more often in cluttered environments. There are two situations to take into account:

- When a dynamic obstacle is occluded.
- When a static obstacle is occluded.

#### 2.2.1. Dynamic Occlusion Detector

When a dynamic obstacle is occluded, we propose the use of a Dynamic Occlusion Detector stage (block 5 in the Figure 1) that estimate the velocities likelihood during the occlusion. This detector is based on the following premises: first the dynamic obstacle can be occluded by other objects (that can be static or dynamic), and it should be farther than object that produces the occlusion. This detector follows the steps showed in Algorithm 1.
**Algorithm 1:** Dynamic obstacle detector algorithm.
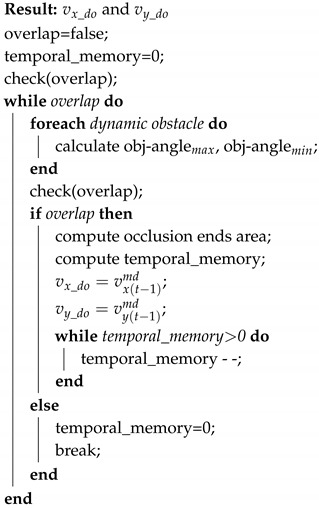


**Figure 4:**
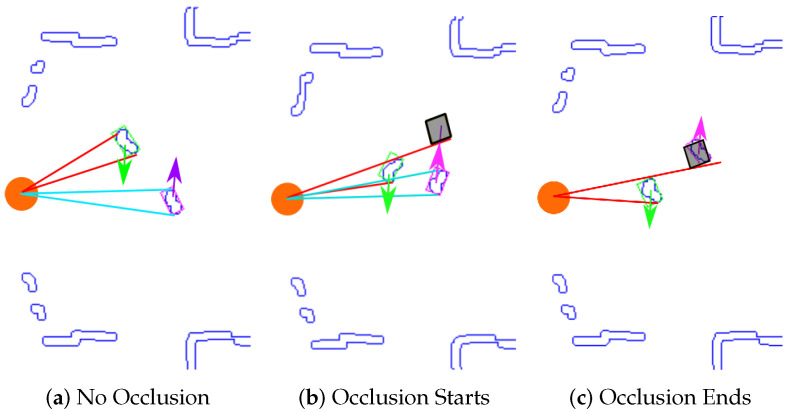
Dynamic Occlusion Detector: different stages.

Figure 4 shows an example of the dynamic obstacle detector performance. For each dynamic obstacle the obj-angle${}_{max}$ and obj-angle${}_{min}$ of the points that form objects respect to the robot are obtained (red lines for the top object and light blue lines for the bottom one in Figure 4a). Occlusion occurs when these two angular sectors begin to overlap themselves (Figure 4b). Once occlusion is detected, the occlusion ends area where the occluded obstacle will probably appear is computed (grey square in Figure 4b,c. This area is obtained in the intersection point between the current velocity vector of the occluded obstacle (purple arrow in Figure 4c) and the angle of the obstacle that produces the occlusion (obj-angle${}_{max}$ or obj-angle${}_{min}$ depending on the case) that it is outside of the overlapped angular sector. In this case it is the upper red line in Figure 4b,c. The occlusion ends area is kept in a temporal memory. It is computed based on the estimated time that the occluded obstacle would take to appear on the other side of the obstacle that produced the occlusion.

The estimated time that the dynamic occlusion lasts (temporal_memory) is computed based on the previous velocities, when the occlusion started ($v_{x\_do}=v_{x(t-1)}^{md},v_{y\_do}=v_{y(t-1)}^{md}$). These velocities ($v_{x\_do}$ and $v_{y\_do}$) are sent to the Tracking Stage in order to update the obstacle prediction (see Figure 1).

#### 2.2.2. Static Occlusion Detector

To avoid losing information about static obstacles occupancy likelihood during an occlusion, we propose to use a Static Occlusion Detector (block 4 in the Figure 1). To estimate the occupancy likelihood of the occluded cell behind an occupied cell (from the point of view of the LIDAR) depends on its previous occupancy and it is obtained following the Algorithm 2.
**Algorithm 2:** Static obstacle detector algorithm.
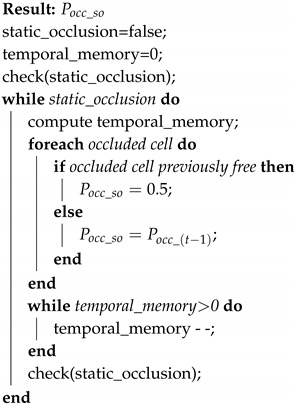


In summary, when a static occlusion is detected and for each occluded cell, if it was previously free, then the occupancy likelihood is set to 0.5 $\left(P_{occ\_so}=0.5\right)$. On the other hand, if the occluded cell was previously occupied, the occupancy likelihood will be kept to the previous value ($P_{occ\_so}=P_{occ\_(t-1)}$). With this algorithm we keep the occupancy likelihood, the estimated time that the occlusion lasts (temporal_memory). In this way, it helps the local planning to take into account previously detected obstacles and it is prepared for a possible unexpected obstacles.

**Figure 5:**
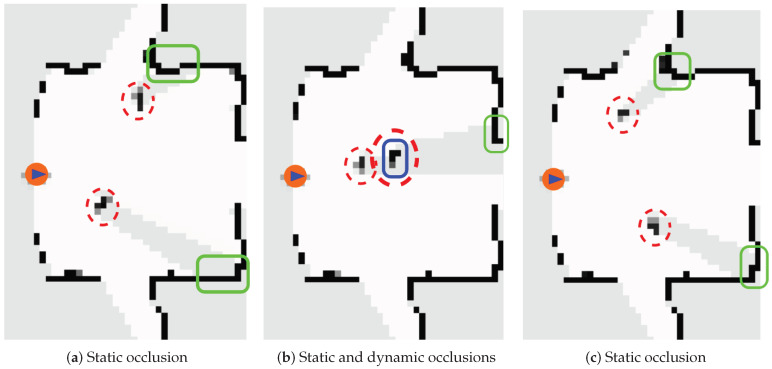
Occlusion Handling: Static and Dynamic obstacles.

Figure 5 shows a real example of the occupancy map (where white represents $P_{occ}=0$ and black $P_{occ}=1$) with two people (marked as dashed-line red circles) crossing in front of the robot at three different time steps. The details of the real scenario will be described in Section 3. Figure 5a,c show how the people occlude parts of the static obstacle (marked with green boxes) and the iDOMap keeps their occupancy avoiding that static objects, such as walls, “disappear” during the occlusion by dynamic objects (this effect is known as spatial memory). Figure 5b shows both kinds of occlusions, the nearest dynamic object occludes the farther dynamic obstacle (marked with the blue box) and, at the same time, some part of the wall (marked with the green box). In both cases the occlusions are handled successfully.

### 2.3. Tracking and Filtering

In the previous versions of DOMap a tracking stage based on Extended Kalman Filter (EKF) was implemented. This method assumed than the dynamic obstacles were differential drive robots which motion model could be linearized between time steps by means of a Jacobian. In order to collaborate with other agents, such people, which motion model is unknown at execution time, in this iDOMap we propose to use a Particle Filter (PF) in the Tracking stage (block 6 in the Figure 1) because in the literature it has been demonstrated to be efficient for this purpose [27,28].

Based on the connectivity of the cells, we can detect a blob (object) that can be useful to track. This tracking stage can manage the noise in measurement and motion models, in a way that helps us to predict the centroid of the object during situations where the rest of the system does not provide enough information, such as the occlusions.

The PF [29] is a Bayesian random-sampling algorithm that allows us to use non-linear motion models assuming that the object dynamics come from a temporal Markov chain. This means that the state ($x_{t}$) is only conditioned by the previous state, independent of the earlier history ($x_{t}=x_{1},...,x_{t}$), allowing quite general dynamics. The PF uses a set of random samples to represent the posterior belief function $bel(x_{t})$. The samples are drawn from the posterior and they are called particles and denoted like:

(1) $$x_{t}^{k}=\left[\begin{array}{c} x^{k}\\ y^{k}\\ \theta^{k} \end{array}\right]$$

where the particle $x_{t}^{k}$ is an instantiation of the state at time *t* and it has an importance factor (weight) denoted $w_{t}^{k}$. This weight describes the importance of particle *k* in the set of *N* particles, denoted:

(2) $$S_{t}=x_{t}^{1},x_{t}^{2},...,x_{t}^{N}$$

The associated importance factors are denoted:

(3) $$W_{t}=w_{t}^{1},w_{t}^{2},...,w_{t}^{N}$$

The likelihood that the hypothesis state $x_{t}$ (centroid in our case) to be included in the particle set $S_{t}$ should be proportional to its posterior $bel(x_{t})$ of its Bayes filter, in the ideal case:

(4) $$x_{t}∼p\left(x_{t}|o_{1:t},u_{1:t}\right)$$

where $o_{1:t}$ are the measurements and $u_{1:t}$ are the control inputs. The denser area of the state space indicates the more likelihood that the true state falls into this area.

The PF algorithm recursively constructs the belief $bel(x_{t})$ from the previous belief $bel(x_{t-1})$. As the particles represent a set of beliefs, the particle set $S_{t}$ is built recursively from the previous set $S_{t-1}$. Hence, Particle Filter is a recursive algorithm that operates in two phases: prediction and update. This means that after each control input, every particle is modified according to the motion model in the prediction stage. For our proposal, the control input is defined as $u_{t}=\left(v_{x}^{md},v_{y}^{md},\Delta \theta \right)$, where $v_{x}^{md}$ is the velocity in X axis and $v_{y}^{md}$ is the velocity in Y axis provided by the Movement Detector stage (block 2 in Figure 1) and Δθ is the incremental orientation change, given by Equation (5)

(5) $$\Delta \theta =atan\left(\frac{\Delta v_{x}^{md}}{\Delta v_{y}^{md}}\right)$$

where $\Delta v_{x}^{md}=v_{x_{t}}^{k}-v_{x_{t-1}}^{k}$ and $\Delta v_{y}^{md}=v_{y_{t}}^{k}-v_{y_{t-1}}^{k}$. The generic motion model is given by Equation (6).

(6) $$x_{t+1}^{k}=\left[\begin{array}{c} x^{k}+v_{x}^{md}\cdot t\\ y^{k}+v_{y}^{md}\cdot t\\ \theta^{k}+\Delta \theta \end{array}\right]+n_{t}$$

where $n_{t}$ is the noise vector.

**Figure 6:**
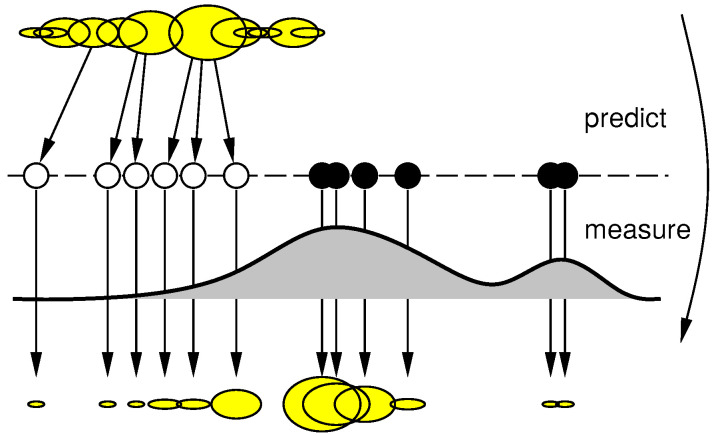
iCONDENSATION Algorithm [32].

After obtaining information from the Movement Detector stage (block 2 in the Figure 1), the particles are weighted based on the Euclidean distance to the blob centroid obtained by theBlob Filtering stage (block 3 in the Figure 1). The PF used is based on iCONDENSATION algorithm by [30]. Also, the samples set size is fixed, in a way that guarantees that the algorithm runs in constant time. This approach is based on the CONDENSATION algorithm that had been extended by the authors adding “Importance Sampling” [31]. This sampling approach improves the efficiency when an auxiliary knowledge (from others sensors) is available, as shown in Figure 6, where the motion model predicts the objects remain on the left (the white samples) and the black samples are positioned based on the auxiliary knowledge that the object has moved to the right. Approximation is more accurate than without this information and robustness to temporary measurements failures is improved. The importance sampling eliminates the low weighted particles and concentrates the filter in particles with high weights.

In the experiments of iDOMap that will be shown in the next sections, there are no other sensors to measure the environment than the LIDAR. However, this feature allows us to fuse measurements from different sources when they are available, improving, in this case, the iDOMap in terms of robustness and accuracy.

In addition, in the velocity estimation stage, a filtering state has been added. A sliding window median filter has been chosen to minimize the effects of the outliers provided by the Movement Detector stage.

## 3. Test Bed and Results

**Figure 7:**
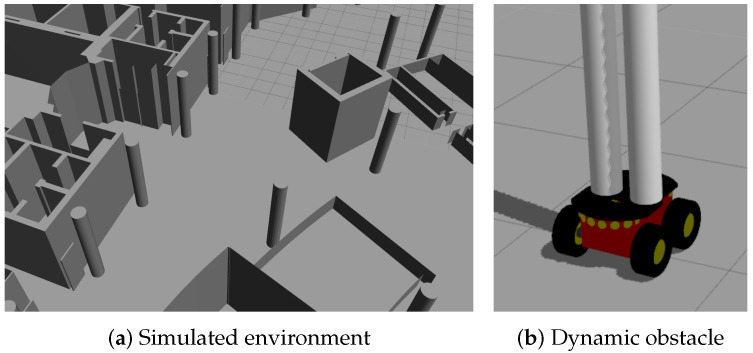
Simulated Test Bed and a robot simulating a person.

To validate our proposal, we performed several simulated experiments before the real ones using Robotic Operating System (ROS) with Gazebo simulator. These experiments demonstrated the feasibility of the proposal and have been performed in a 3D map of the Polytechnic School (Figure 7a). In this environment there will be dynamic obstacles moving around the robot. These dynamic obstacles have been simulated with other robots, in order to control the velocities and the paths that they follow and to obtain, in this way, the Ground Truth for the simulated results. In addition, in order to simulate the legs of a person, some robots have two vertical cylinders above the platform (Figure 7b).

**Figure 8:**
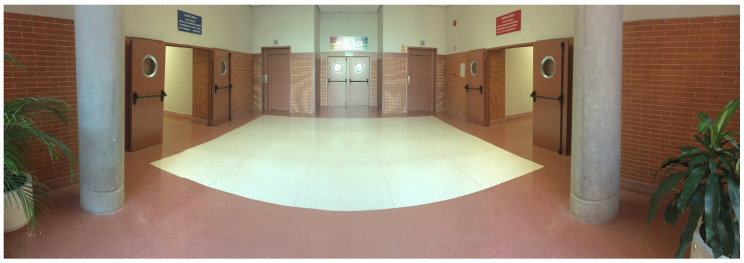
Real Test Bed: Indoor scenario (Robot point of view).

The real system was tested in a controlled environment inside the Polytechnic School to avoid problems with the estimation of our own velocity and possible slips. Testing area is approximately 7 × 10 m, where there are three doors and two lifts (Figure 8). The hardware used in the real experiment has been our own robotic platform RoboShop equipped with a Brix i7 with Ubuntu and a LIDAR SICK LMS 151.

Although the proposed method is a Grid-Based approach and the output should be an occupancy grid with velocities and labels associated with each cell, as it is shown in the bottom part of the iDOMap diagram (Figure 1), the results will show the data at the “object level” in order to clarify the explanation.

Obtaining the Ground Truth in real experiments is always a big challenge, more over when there are agents in the environment that cannot provide information about their own states, such as people, animals, etc. In DATMO systems there are several approaches; in outdoors environments each dynamic obstacles can carry out a Global Positioning System (GPS) as the authors proposed in [33], other work considered that in the presence of moving objects without sensors any system will have a difficult time assessing the accuracy of tracking data [34]. For that reason some authors only report qualitative results, such as “all the dynamic obstacles are well detected with an accurate velocity” in [35]. On the other hand, other authors proposed an affordable approach for people tracking Ground Truth, based on markers in the ground, where the people are walking and a stop-watch to measure the time between marks as in [36] (called visual marked Ground Truth).

**Figure 9:**
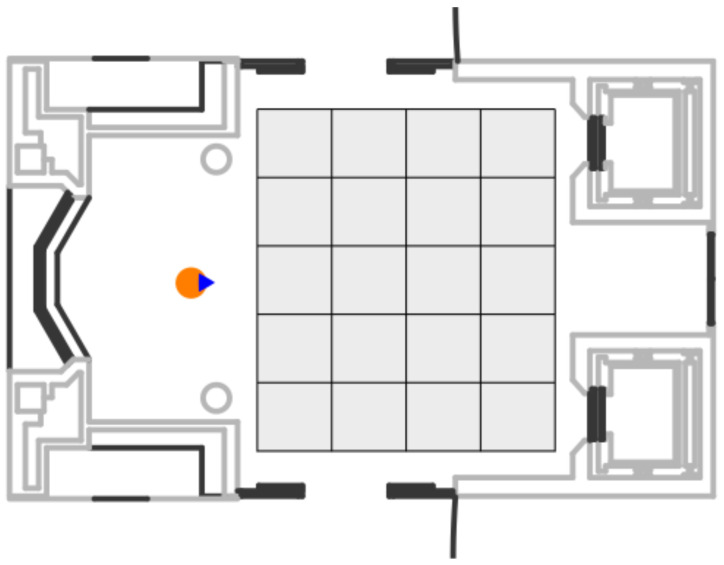
Ground Truth: Indoor scenario.

In this paper, a Ground Truth based on this idea was performed. A video of the scene is recorded synchronized with the LIDAR measurements of the robot using ROS timestamps. The dynamic obstacles move in the area that is recorded by the camera where a grid in the floor (grey grid in Figure 9) is present. Then, hand-made frames are selected when the obstacles are in key points of the grid. Dimensions of the floor grid are well known and a constant velocity between these points is assumed. A more accurate Ground Truth is beyond the scope of this paper.

The desirable Ground Truth would be an intelligent environment, big enough to perform crossing maneuvers and able to detect the pose and velocities of all participants in the scene. Due to the lack of this kind of Ground Truth system and in order to measure the position and angle of the dynamic obstacles at the highest possible accuracy, the robot (orange circle in Figure 9) was placed in a well-known and static position respect to the floor grid.

### 3.1. Velocities Estimation with iDOMap

The simulated experiments have been performed to know when and where exactly our robot and the participants are. This complete knowledge over the scenario allows us to obtain results while the robot is moving in the environment. All scenarios have been tested simulation and in real mode and due to all of them obtained similar results to the real ones and in order to reduce the length of this work, we are going to show only the first simulated scenario.

**Figure 10:**
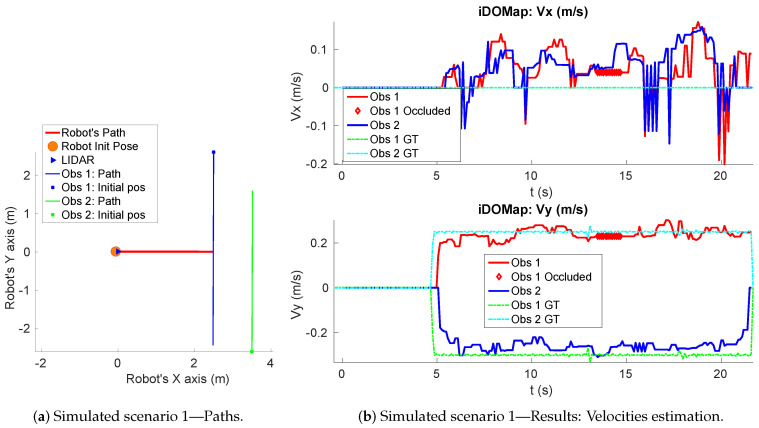
Simulated scenario 1—Paths and Velocities estimation.

- **Simulated scenario 1—Two dynamic obstacles crossing:** In this scenario there are two dynamic obstacles simulated persons crossing in perpendicular paths in front of the robot at 0.25 m/s and 0.3 m/s. The paths followed by the obstacles are shown in Figure 10a. This scenario shows the performance of the proposal across the Yaxis of the robot at low velocities.
  Figure 10b shows the velocities detection at each axis compared with the Ground Truth. Table 1 shows the errors in velocities detection where it can be seen that the error is around 0.05 m/s at each axis.

Because one of the objectives of this paper is to get a safe interaction between humans and robots, it is interesting to test the estimation of velocities with iDOMap in a real scenario with people who cause dynamic occlusions. As the Ground Truth is limited to the camera Field Of View (FOV), in some experiments, it ends before than the output of proposal system, due to the laser FOV is wider than the camera’s one. As mentioned before, in those experiments the robot has been placed in a static position while the obstacles are moving in front of it. Experiments with the robot in movement will be shown in next section.

Although at the beginning of each scenario, the people could be enumerated to introduce the scenarios, in the next paragraphs will be denoted as dynamic obstacles or objects because the proposal do not take into account which kind of dynamic obstacle they are. The results are in the robot coordinate system, where the positive velocity on the X axis denotes moving away from the robot and the positive velocity on the Y axis denotes a movement from the right to the left of the robot.

**Figure 11:**
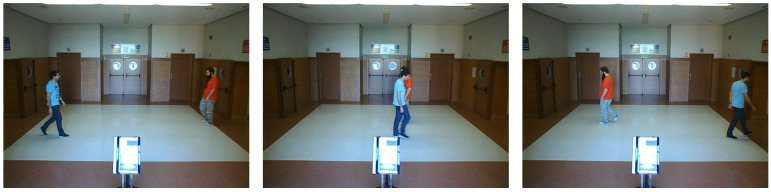
Real scenario 1—Images sequence.

**Figure 12:**
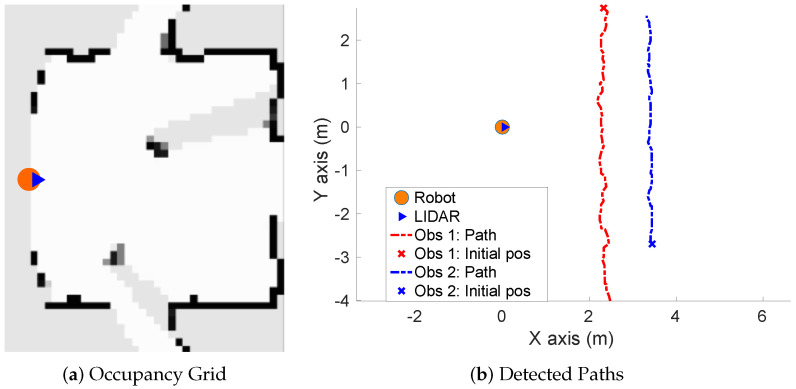
Real scenario 1—Occupancy Grid and Paths detected.

**Figure 13:**
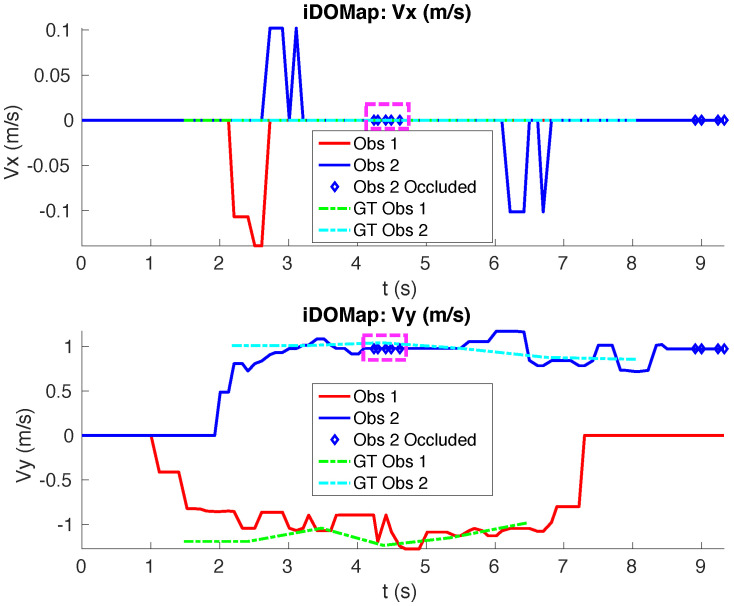
Real scenario 1—Detected velocities.

**Figure 14:**
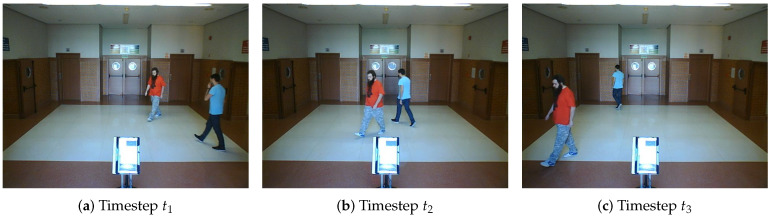
Real scenario 2—Images sequence.

**Figure 15:**
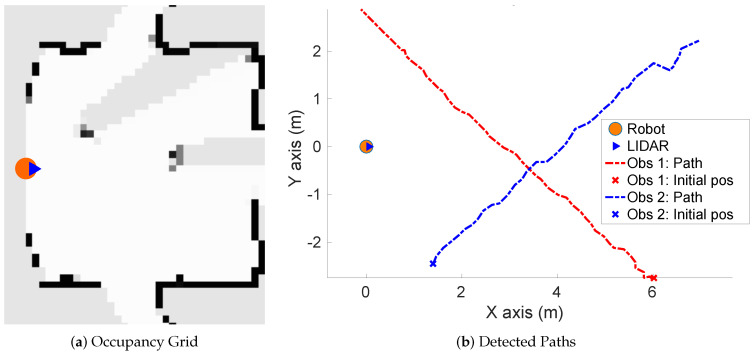
Real scenario 2—Occupancy Grid and Paths detected.

**Figure 16:**
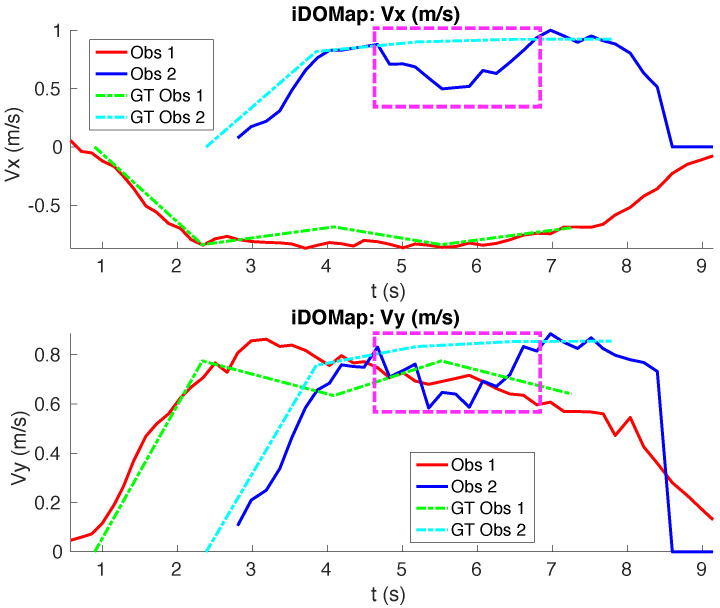
Real scenario 2—Detected velocities.

**Figure 17:**
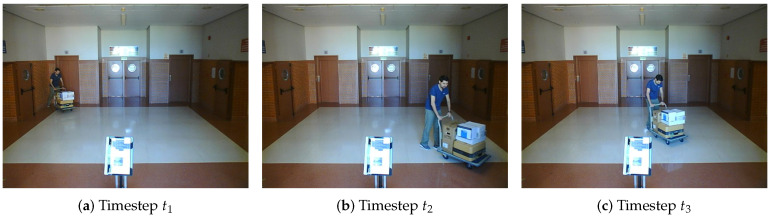
Real scenario 3—Images sequence.

**Figure 18:**
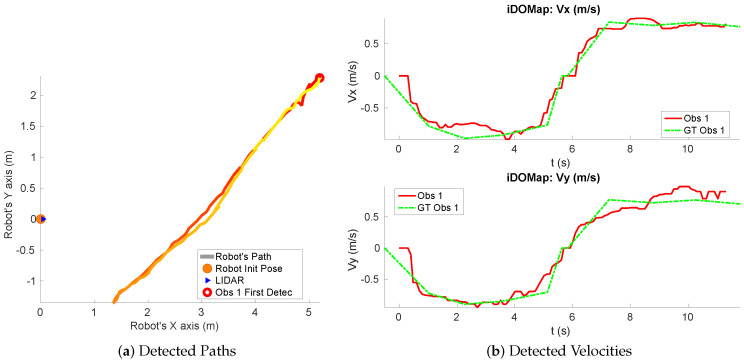
Real scenario 3—Path and detected velocities.

**Figure 19:**
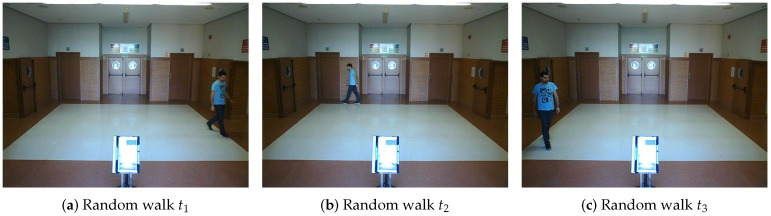
Real scenario 4—Images sequence.

**Figure 20:**
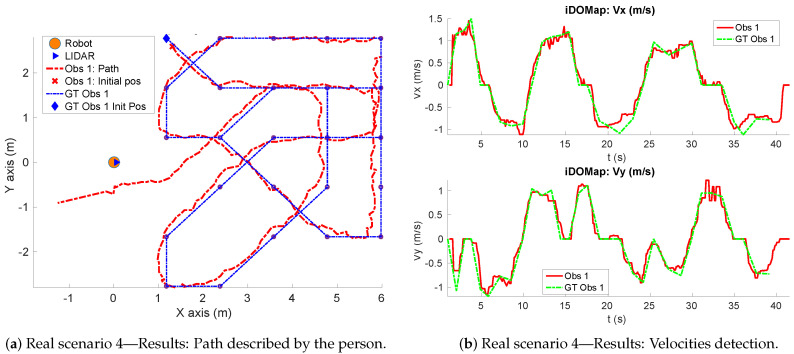
Real scenario 4—Path and Velocities detection.

**Figure 21:**
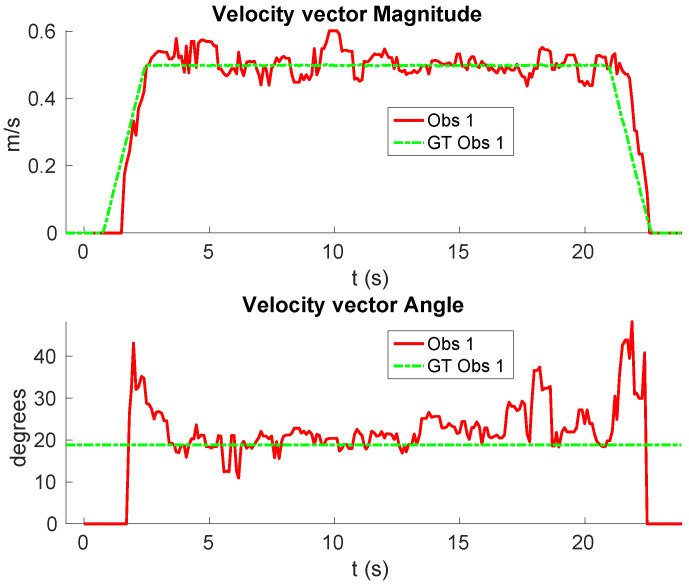
Real scenario 5—Results: Angle and Magnitude vectors.

- **Real scenario 1—Two dynamic obstacles crossing:** In this scenario there are two people crossing perpendicularly to the robot and then obstacle 1 (which starts on the left) causes an occlusion over the obstacle 2, as can be shown in timestep $t_{2}$ in Figure 11b. Figure 11 shows images at three time steps during the experiments while the robot detects and tracks the paths followed by dynamic obstacles (Figure 12b).
  In addition, an occupancy grid map of this scenario during the experiment (Timestep $t_{3}$) is shown in Figure 12a. In this occupancy grid, it can be seen as the cells occupied by the static and dynamic obstacles have high occupancy probability (the darker the higher is the probability). Figure 13 shows the velocities detected at each axis of both obstacles, where it can be seen that the velocities of the obstacle 2 are kept during the occlusion (marked as purple dashed-line box).
  Table 2 shows the errors in the velocities detection for this scenario, where $\bar{\epsilon }$ is the average error and $\sigma_{\epsilon }$ the standard deviation of the following parameters: Vx and Vy are the obstacle velocities along Xaxis and Yaxis of the robot respectively, ||V|| is the vector velocity magnitude and θ the vector angle.
- **Real scenario 2—Two dynamic obstacles in diagonal paths:** In this scenario, there are two people crossing in diagonal paths, as shown in Figure 14. Each path crosses the other as shown in Figure 15b. Figure 15a shows an example of the occupancy grid computed during this experiment.
  The detection of the obstacle 2 worsens between seconds 5 and 6 (marked as purple dashed-line box in Figure 16) due to the low amount of laser impacts. This situation appears when the obstacle (a person in this scenario) is located laterally to the laser.
  Table 3 shows the averages velocity errors ($\bar{\epsilon }$) and their standard deviation (σ) for each axis.
- **Real scenario 3—Person pushing a trolley in diagonal path:** As stated previously, robots and people can coexist at the same environment, then they can be pushing objects, such as baby carriage or shopping cart. In order to test our proposal with this kind of dynamic obstacles, a person pushing a trolley performed two diagonal paths in forth and backward ways. The scenario can be seen in Figure 17.
  Figure 18a shows the detected paths followed by the person, in this case, the red path shows the forward path and the yellow one the backward path. Figure 18b shows the velocities detection at each axis. It can be seen that both paths (forwards and backwards) have been detected with an errors around 0.1 m/s, as it can be shown in Table 4.
- **Real scenario 4—Person walking randomly:** In this scenario a participant is walking in different directions inside of the monitored area and making strong changes in direction. With this experiment we demonstrate that PF is able to deal with a real movement of a person that cannot be followed by an EKF, as it can be seen in Figure 19.
  This scenario has been selected to test the performance with several direction changes in a short period of time.
  Figure 20a shows the path detected by our proposal, where it can be seen that despite the frequent and rapid changes of direction that the obstacle describes, the system can detect the velocity at each axis with an error less than 0.1 m/s, as it is shown in Table 5. Also the position tracked (shown in red in Figure 20b) follows in a good manner the Ground Truth (shown in blue).
- **Real scenario 5—A robot crossing in a diagonal path:** Continuing with the idea of test our proposal with different participants, in this scenario, a robot crosses in a diagonal path with a constant linear velocity of 0.5 m/s and in an angle of $18^{\circ }$ respect to the robot with iDOMap. The Ground Truth in this case has been obtained by taking into account the initial and final static positions, because the obstacles maintain this position for 5 s. Then, hand-made centroid has been computed from the measures of LIDAR during the obstacle’s static period of time.
  Figure 21 shows the vector magnitude ||V|| and angle (θ) of the velocity detection in order to clarifying the comparison with the Ground Truth. Table 6 shows the error in velocities detections, where it can be seen that the error velocity magnitude vector is lower than 0.04 m/s and the error in orientation is about $6^{\circ }$.
  The vector velocity magnitude percentage error ${\bar{\epsilon }}_{\left||V|\right|}$ and $\sigma_{\epsilon }$ are the highest experimental errors, because when the Ground Truth velocities are very low (when the obstacle starts and ends move), the error detected (in percentage) is very high. In this case, if only dynamic obstacles are taking into account when their velocities are upper a threshold (0.15 m/s) the error would be ${\bar{\epsilon }}_{\left||V|\right|}=7.67\%$ and $\sigma_{\epsilon }=15.20\%$.

In summary, we have tested our method in simulated and real scenarios, with several kinds of participants and velocities up to 1.5 m/s, and the error in velocity magnitude vector (${\bar{\epsilon }}_{\left||V|\right|}$) remained lower than 14%. In a few cases, when the obstacles were thin and were located laterally to the LIDAR, the errors increased up to 21%. In addition, the error in orientation remained lower than $13^{\circ }$. Taking into account the low speeds that has been handled in the experiments, the results obtained (in velocities and orientation) with the proposal are able to increase the obstacle-avoidance algorithms performance as will be shown in the next section.

### 3.2. Testing the Improvement of Obstacle-Avoidance Algorithms Using iDOMap

In this section, and in order to show the improvement of the obstacles avoidance systems when the iDOMap is used, some real experiments have been performed in different scenarios with several number of obstacles. At each scenario the system has been tested including the new iDOMap and without it, which means taking the raw impacts laser data as local mapping.

Classical obstacle-avoidance algorithms evaluated in our previous work by [37] were able to deal with dynamic obstacles if the movements of the robot are significantly faster than the dynamic obstacles. However, avoiding them like static ones can result in non-optimal trajectories and risky situations, especially if the measurements of the environment are not accurate enough or even the obstacles’ direction suddenly change. To take into account these matters, in our previous work [15] we proposed two algorithms that deals with dynamic obstacles.

In this section, we test the improvement of using iDOMap in Dynamic Curvature Velocity Method or DCVM [37] and Dynamic Window for Dynamic Obstacles or DW4DO [15] avoidance algorithms.

#### 3.2.1. Simulated Experiments

**Figure 22:**
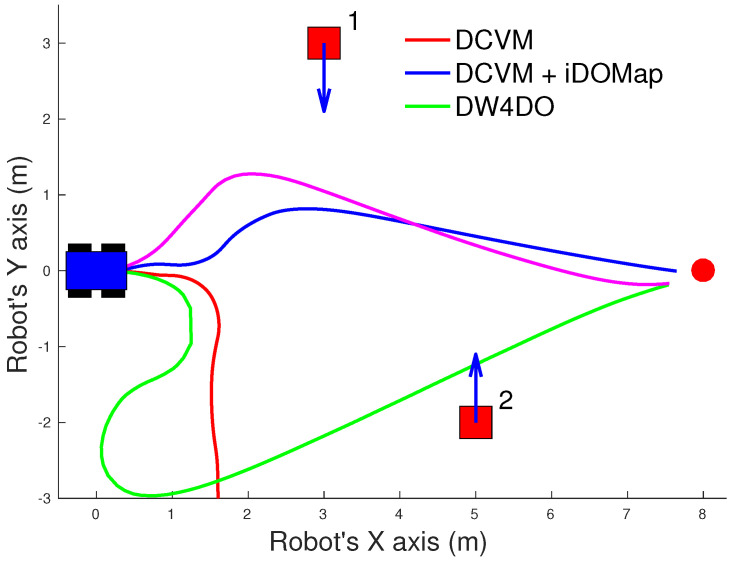
Scenario 1: Paths Results.

**Figure 23:**
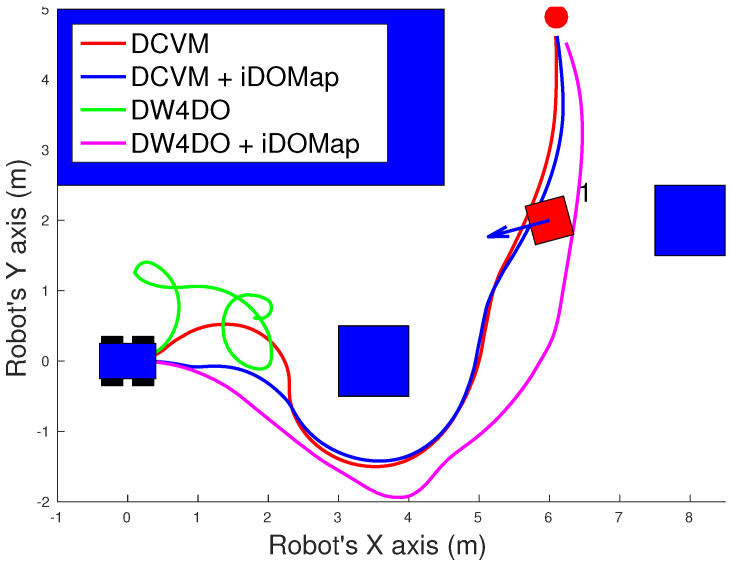
Scenario 2: Paths Results.

**Figure 24:**
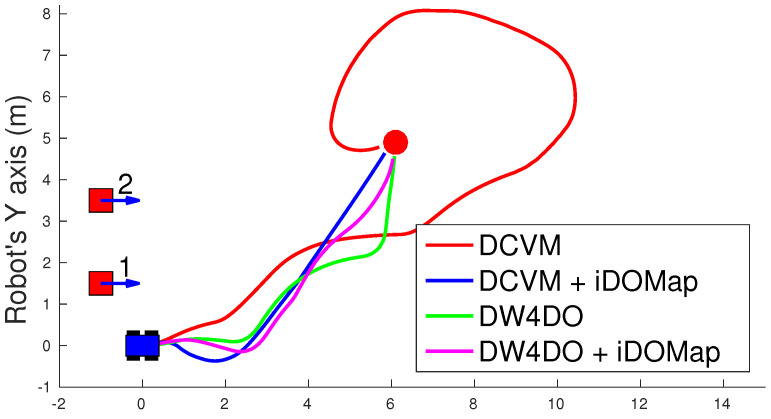
Scenario 3: Paths Results.

In these simulated experiments the moving obstacles has been simulated with other robot platforms to know their velocities and pose. Each scenario has been performed with the two obstacles avoidance algorithms introduced in Section 3.2 with raw LIDAR data and with the perception stage proposed. The system has been simulated in the scenarios as follow:

- **Scenario 1—Two obstacles crossing:** in this scenario, the final goal is in front of the robot and two objects cross in perpendicular paths to the robot at 0.4 m/s. Figure 22 shows the starting position and orientation of the obstacles. Also it shows the path followed by the robot at each case. This scenario tries to simulate a risky environment where the robot could cross the trajectories of the moving obstacles.
  The paths followed (Figure 22) show that without knowledge of the obstacles velocities, both algorithms try to overcome the obstacles crossing in front of them, and the obstacles block the possible paths to the goal making the robot travels parallel to the first obstacle until this obstacle is far enough away to go to the goal. On the other hand, knowing the estimated velocities of the obstacles, both avoidance algorithms try to go to the goal overcoming the obstacle behind them in a safer maneuver. Also, these two maneuvers are more efficiency, due they are shorter in distance and time, as it is shown in Table 7.
- **Scenario 2—Moving obstacle approaching the robot:** this scenario is a more complex environment that mixes static and dynamic obstacles (Figure 23). Two static obstacles (blue squares) have been placed: a corridor has been formed between the big obstacle (on the left of the robot) and the obstacle in front of it. Also, another obstacle has been placed in the corner that forms another wider corridor. In addition there is one moving obstacle (red square) approaching to the robot in collision path to it through the first corridor. Therefore, a corridor that will be blocked by a moving obstacle has been simulated. This scenario has been selected to deal with this risky situation when the robot could be locked in a small space due to an obstacle is moving to the robot (local minimum).
  Figure 23 shows the static obstacles (blue squares) and the starting position and orientation of the dynamic obstacle (red square). Also, Figure 23 shows how without knowledge about the obstacle velocity, the DCVM tries to enter to the narrow corridor to later change the direction when the obstacles block its path. Even worst is the case of the DW4DO due to without a prediction module is not prepared to react to dynamic environments, computing the shortest path in every time step and causing loops trajectories. On the other hand, with our proposal, both algorithms can reach the goal knowing that the narrow corridor will be blocked and avoiding this path. The paths followed with iDOMap are safer and more efficient than without it due to are shorter in time and distance as can be seen in Table 7.
- **Scenario 3—Lane changing:** In this scenario moving obstacles are travelling in the same direction as the robot, so it simulates a lane changing in a road (see Figure 24). The obstacle nearer the robot travels at 0.75 m/s and the further one at 0.4 m/s.
  This scenario is similar to the previous one in terms of riskiness, due to the robot could cross the dynamic obstacles trajectories. In the case of only take into account the raw LIDAR measurements, the DCVM spends a lot of time trying to overcome the obstacle travelling parallel to them. This situation occurs when the velocities of the obstacle and robot are similar, until the obstacle is far enough to avoid it. Similar is the case of the DW4DO without our proposed iDOMap. However, if the algorithm has the velocities estimation, both of them begin to separate from the nearer obstacle (because it is safer) and go to goal crossing behind both obstacles in a safer and smoother path, as shown by the time and distance travelled data for these cases in Table 7.

A summary of paths followed by all the combinations of both obstacle-avoidance algorithms exposed in Section 3.2.1, with LIDAR raw data and iDOMap as perception stages, it is shown in Table 7. The parameters shown in Table 7 measure velocity of the maneuver: distance travelled d(m) and time spent t(s) to reach the goal, also the medium linear velocity $\bar{v}\left(m/s\right)$, while others measure the smoothness of the path: medium absolute angular velocity $|\bar{\omega }|{(}^{\circ }/s)$ and the velocities standard deviation: $\sigma_{v}^{2}$ and $\sigma_{\omega }^{2}$. Taking into account these parameters, it can be shown how the distance travelled and the time spent had been reduced in the three scenarios with our iDOMap. In addition, when our perception stage is available, the $\sigma^{2}$ is lower, meaning that smoother paths are followed. Also, in some risky situations, without velocities estimations, the goal cannot be reach or the path followed is huge, as the case of DCVM in the Scenario 1 and the DW4DO in the Scenario 2. For that reason, taking into account the velocities estimation provided by our proposal reduces the time spent and the distance travelled to reach the goal, moreover the performed path is smoother and safer.

#### 3.2.2. Real Experiments

**Figure 25:**
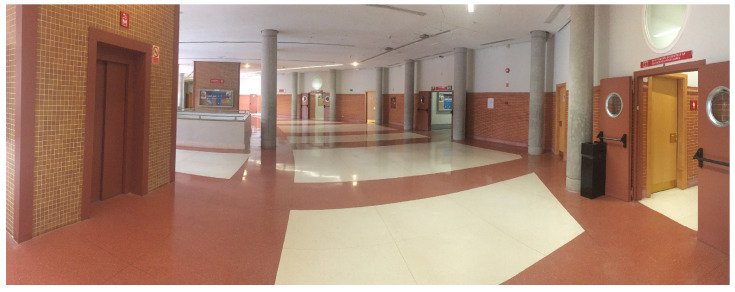
Test Bed: Indoor scenario.

Experiments were performed in a controlled indoor environment in the Polytechnic School at the University of Alcalá (UAH). The area is 6 × 12 m approximately, where there are several doors, corridors and columns (Figure 25). The dynamic obstacles were people, therefore the exact positions and velocities cannot be controlled in an accurate way, increasing the difficulty to make repeatable and comparable real scenarios. First we checked the proposal in simulation to evaluate the improvement, and then we compared the real results obtaining similar results.

**Figure 26:**
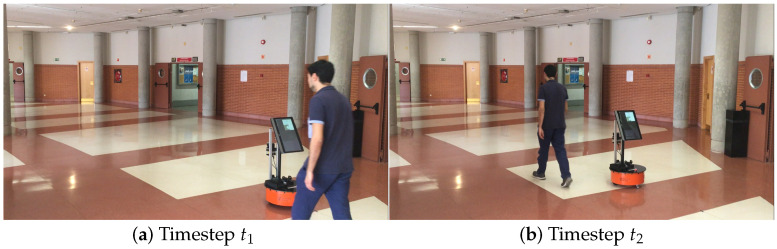
Scenario 1—Images sequence.

**Figure 27:**
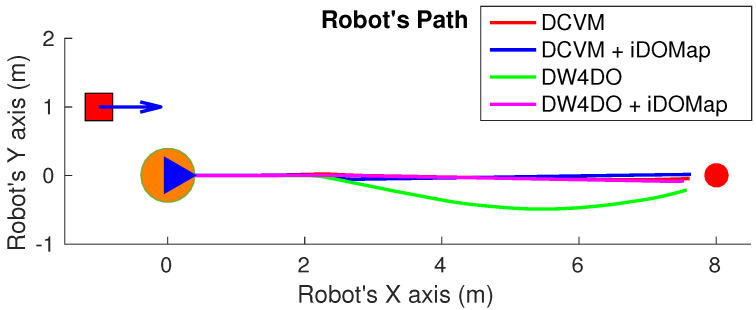
Scenario 1—Robot’s Paths.

**Figure 28:**
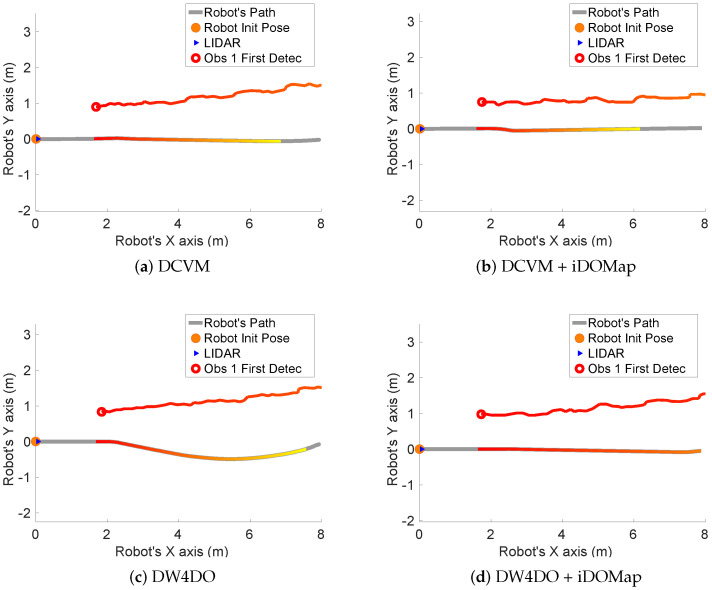
Scenario 1—Robot Paths and iDOMap Detections.

**Figure 29:**
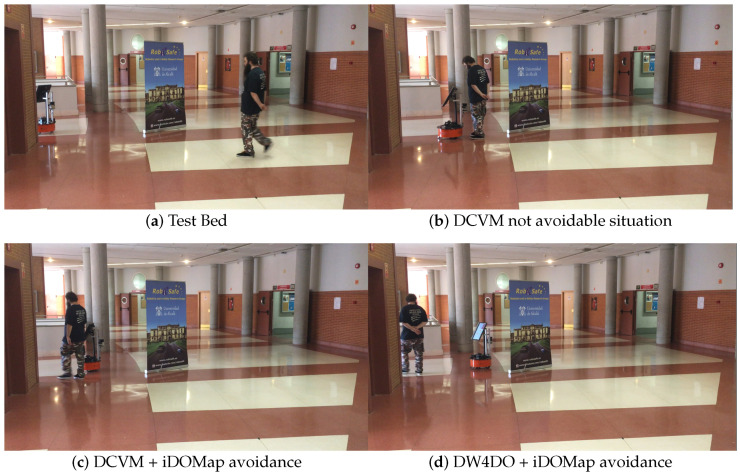
Scenario 2—Images sequence.

**Figure 30:**
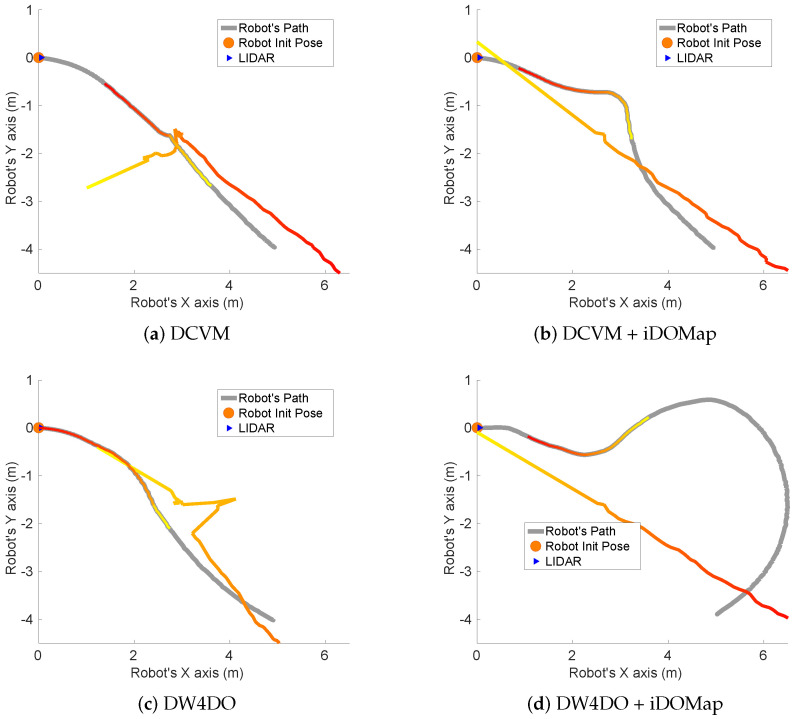
Scenario 2—Robot Paths and iDOMap Detections.

**Figure 31:**
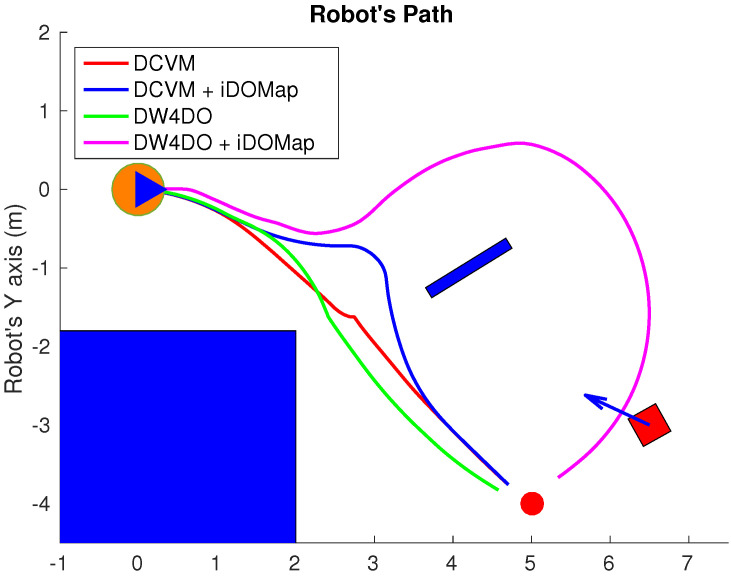
Scenario 2—Robot’s Paths.

**Figure 32:**
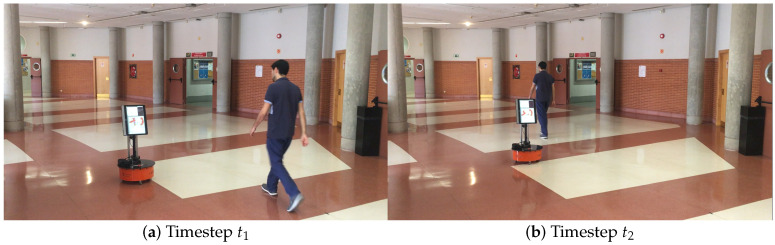
Scenario 3—Images sequence.

**Figure 33:**
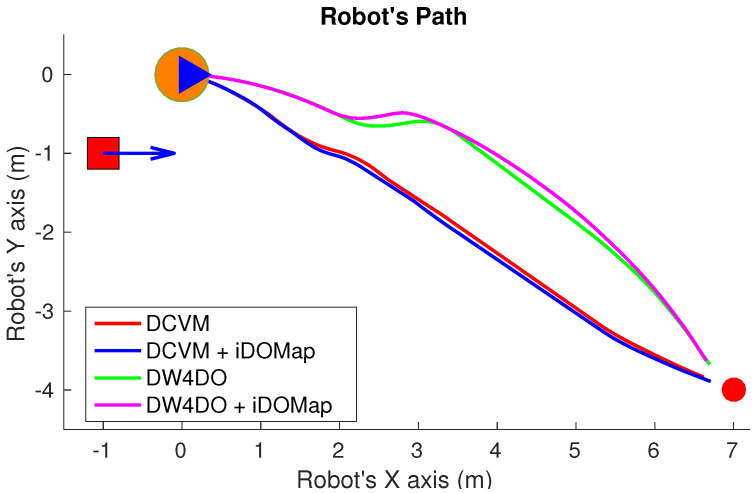
Scenario 3—Robot’s Paths.

**Figure 34:**
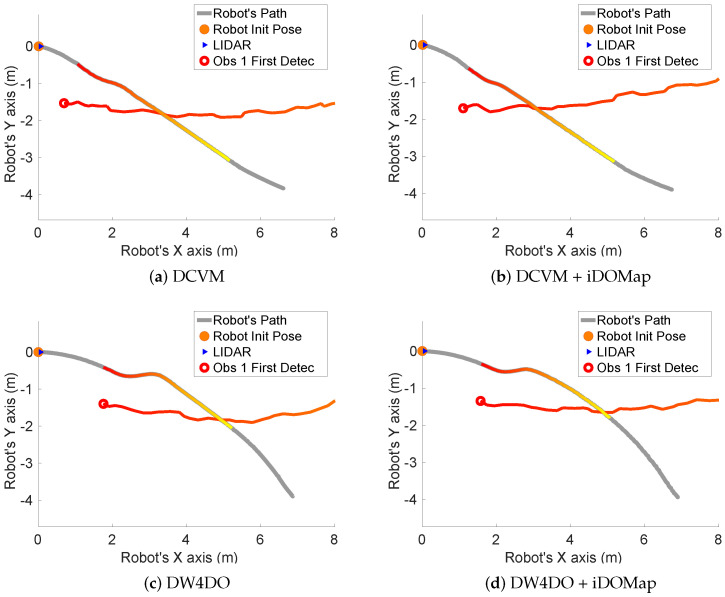
Scenario 3—Robot Paths and iDOMap Detections.

Taking these constraints into account, these real scenarios have been performed:

- **Scenario 1—A person overtaking the robot:** In this scenario (see images sequence in Figure 26), the robot has to reach a goal 8 m forward from its initial position (red dot in Figure 27), while a person (marked as red square in Figure 27) overtakes the robot by the left.
  The paths followed by the robot at each combination of perception and obstacles avoidance algorithm are shown in Figure 27. Figure 28 shows the detected dynamic obstacle at each case, where the grey line represents the robot path. The color of the obstacle path and the robot path match (from red to yellow) during the period of time that our method detects the obstacle, in order to show them in a synchronized way.
  Figure 28 has been limited to 8m, in order to show this clearly, although the obstacle has continuously been detected.
  We evaluate the four cases in the same way, and assuming that they can be compared. When an estimated velocity is introduced in the case of the DCVM, the robot moves away from its path compared to the case without velocity estimation (Figure 28a), increasing the safety. Figure 28c,d shows other behavior performed by DW4DO; without velocities estimation (Figure 28c) when the obstacle appears in the robot map near the robot, suddenly it tries to move away of the obstacle, on the contrary, if iDOMap is available (Figure 28d), the velocities of the obstacle are estimated, then the algorithm estimated that the overtaking of the dynamic obstacle do not influence in its trajectory, keeping the direction, increasing the energy efficiency of the path due to it has fewer changes in its velocities and a shorter travelled path.
- **Scenario 2—Moving obstacle approaching the robot:** This scenario has been selected in order to test a risky situation when the robot could be locked in a small space, between the wall in left part of Figure 29a and the auxiliary wall located in the middle of Figure 29a, due to an obstacle is moving to the robot (local minimum).
  Figure 30 shows the detected dynamic obstacle in each case, where the grey line represents the robot path. The color of the obstacle path and the robot path match (from red to yellow) during the period of time that our proposal detects the obstacle in order to show in a synchronized way the pose of the robot when the obstacle is detected at each step. Figure 30 has been limited to maneuver area, in order to show it in a clarifying way, for that reason the obstacle first detection is outside of the figure.
  Figure 31 shows the behavior of both algorithm without velocities estimation and with our proposal. In the case of the DCVM when the velocities are not available, the robot tries to pass through the corridor and when the obstacles “appear” (Figure 29b) is too late to avoid it, then the robot stops and spins (position 3, −1.5 m approx) being a risky situation. That spins maneuver and that the obstacle is too close to the robot, making the detection inaccurate at this point (Figure 30a. When iDOMap is combined with the DCVM (blue path) the robot avoids it to the left (Figure 29c), reducing its velocity and then continue to the goal. In the case of the DW4DO without our proposal is similar to the DCVM due to is a “not avoidable situation” in both cases. On the contrary, with DW4DO and iDOMap the algorithm is able to predict that the obstacle will be blocking the pass through the corridor, avoiding this situation (Figure 29d) an reaching the goal in a longer but safer path (pink path).
- **Scenario 3—Lane changing:** In this scenario (see Figure 32), the robot has to reach a goal that is at 7 m forward and 4 m to right from its initial position (red dot in Figure 33), during the manoeuvre a person (marked as red square in Figure 33) overtakes the robot in a parallel path by the right.
  The paths followed by the robot at each combination of perception and obstacles avoidance algorithm are shown in Figure 33. Figure 34 shows the detected dynamic obstacle at each case, where the grey line represents the robot path. The color of the obstacle path and the robot path match (from red to yellow) during the period of time that our system detects the obstacle. This shows where the robot and the obstacle were at a particular point in time.
  Figure 34 has been limited to 8m, to improve clarity, although the obstacle has continuously been detected.
  We evaluated four cases in the same way. Figure 33 shows that both obstacle-avoidance algorithms move away from the obstacle, DCVM to the right and DW4DO to the left. Both methods produce safer manoeuvers with smoother velocity profiles when using iDOMap.

Although the results are not comparable at the same level, because they are not completely replicable, Table 8 shows the numerical results in the same way than Table 7 in order to give an idea of the performance. In the Scenario 1 it can be seen that the case of DW4DO with iDOMap spends less time (18.96 s) and travels less distance (7.52 m) and also follows a smoother path (lower $|\bar{\omega }|$ and $\sigma_{\omega }^{2}$) because it keeps its trajectory as has been explained before. In the Scenario 2, when iDOMap is present, both obstacles avoidance algorithms travel longer paths, but in a safer way, overcome “Stop&Go” maneuvers (as its shown in Figure 30a) and the risky situation with erratic movements in Figure 30c. Finally, in the Scenario 3, although the numerical results are very similar, in Figure 33 it can be seen that using our method, the paths are safer, because the avoidance manoeuvre starts earlier.

These real experiments show that including the proposed iDOMap increases the performance of obstacle-avoidance algorithms, helping to overcome “near-collision situations”. These situations are more likely when the robot does not take into account the dynamics of the obstacles. Using iDOMap we obtain and improvement in the safety of the paths and in other cases reduces the energy consumption.

## 4. Conclusions and Future Works

The goal of this paper is to develop an improved version of our previous DOMap by means of using three key points: using laser reflectivity in the movement detection stage, adding static and dynamic occlusion detectors and using Particle Filter instead an Extended Kalman Filter in the tracking stage to deal with people as dynamic obstacles.

The movement detection stage is based on optical flow applied to the laser measurements to detect the obstacle velocities. In order to improve the performance of this algorithm, the reflectivity of the measurement has been included to get a better points matching taking into account more features than only the positions of the impact points.

Occlusion detectors have been added to the system to deal with two kind of situations: when static or dynamic obstacles are occluded. Using the concept of temporal_memory we keep an occupancy estimation for static and dynamic objects during the time that occlusion happens, improving the next tracking stage.

In addition, a PF algorithm has been used in the tracking stage instead of EKF to deal with people as dynamic obstacles. In this way the tracker can deal with strong changes in direction of people’s movement that cannot be followed by an EKF.

The iDOMap has been tested previously in simulation and then in real scenarios to check the velocities estimation with occlusions and to verify the improvement with two of our latest obstacle-avoidance algorithms. The objective of these tests has been to show how the velocities detection of the dynamic obstacles improves the performance of the obstacle-avoidance algorithm, especially in risky situations, resulting more safety and energy efficient paths.

In a close future we propose to extend our iDOMap for using inside Unmanned Aerial Vehicles (UAV). For this purpose, occupancy grid should add a new coordinate, obtaining a 3D occupancy grid, and LIDAR should be also a 3D LIDAR for estimating velocities in all axes (X, Y, Z). In order to reduce the computation an access to the occupancy data, especially needed when we work with UAV, Octrees-based map [38] would be recommended. To our knowledge only a few works exist that use LIDAR reflection for object identification tasks. The authors of [39] use LIDAR reflection for point cloud segmentation based on the diffuse and specular reflection behaviors. In work [40] authors detect traffic lanes on roads by exploiting the LIDAR reflection of lane marking. In [41] they use top view LIDAR elevation and reflection data for road detection. Although most of the 3D-LIDAR-based vehicle detection systems were built on range data, we will plan as future work to use 3D-LIDAR reflection intensity data for vehicle detection. Our approach will be to generate a dense reflection map from the projected sparse LIDAR reflectance intensity and inputted to a Deep Convolutional Neural Network (YOLO) object detection framework for the objects identification.

Although the proposal has been tested in different simulated and real environments with different platforms, expanding our system to 3D it would be interesting to test it in other environments, with higher number of obstacles and other robotic platforms in order to test the performance.

## Figures and Tables

**Table 1:** Simulated scenario 1—Errors in velocities detection.

		$V_{x}$ (m/s)	$V_{y}$ (m/s)
Obs 1	**$\bar{\epsilon }$**	0.051	0.038
	**$\sigma_{\epsilon }$**	0.045	0.049
Obs 2	**$\bar{\epsilon }$**	0.045	0.021
	**$\sigma_{\epsilon }$**	0.047	0.030

**Table 2:** Real scenario 1—Errors in velocities detection.

		$V_{x}$ (m/s)	$V_{y}$ (m/s)	||V|| (m/s)	θ ${(}^{\circ })$
Obs 1	**$\bar{\epsilon }$**	0.011	0.153	0.152 (13.12%)	0.71
	**$\sigma_{\epsilon }$**	0.036	0.127	0.127 (10.70%)	2.19
Obs 2	**$\bar{\epsilon }$**	0.0137	0.092	0.092 (9.75%)	0.79
	**$\sigma_{\epsilon }$**	0.035	0.075	0.075 (8.12%)	2.05

**Table 3:** Real scenario 2—Errors in velocities detection.

		$V_{x}$ (m/s)	$V_{y}$ (m/s)	||V|| (m/s)	θ ${(}^{\circ })$
Obs 1	**$\bar{\epsilon }$**	0.056	0.073	0.078 (12.19%)	7.44
	**$\sigma_{\epsilon }$**	0.041	0.045	0.063 (17.88%)	10.57
Obs 2	**$\bar{\epsilon }$**	0.144	0.098	0.170 (17.86%)	5.10
	**$\sigma_{\epsilon }$**	0.130	0.072	0.141 (16.21%)	5.87

**Table 4:** Real scenario 3—Errors in velocities detection.

		$V_{x}$ (m/s)	$V_{y}$ (m/s)	||V|| (m/s)	θ ${(}^{\circ })$
Obs 1	**$\bar{\epsilon }$**	0.090	0.102	0.104 (21.01%)	13.26
	**$\sigma_{\epsilon }$**	0.077	0.083	0.097 (75.3%)	20.82

**Table 5:** Real scenario 4—Errors in velocities detection.

		$V_{x}$ (m/s)	$V_{y}$ (m/s)	||V|| (m/s)	θ ${(}^{\circ })$
Obs 1	**$\bar{\epsilon }$**	0.111	0.100	0.144 (14.31%)	11.58
	**$\sigma_{\epsilon }$**	0.090	0.095	0.117 (13.34%)	21.98

**Table 6:** Real scenario 5—Errors in velocities detection.

		$V_{x}$ (m/s)	$V_{y}$ (m/s)	||V|| (m/s)	θ ${(}^{\circ })$
Obs 1	**$\bar{\epsilon }$**	0.037	0.033	0.031 (30.65%)	6.10
	**$\sigma_{\epsilon }$**	0.039	0.033	0.038 (229.40%)	5.81

**Table 7:** Simulated experiments: Results.

Experiment	Algorithm	d	t	$\bar{v}$	$\sigma_{v}^{2}$	$\bar{\left|\omega \right|}$	$\sigma_{\omega }^{2}$
Scenario 1: Two Obstacles Crossing	DCVM	–	–	–	–	–	–
	DCVM + iDOMap	7.90	20.28	0.38	0.009	7.93	12.88
	DW4DO	12.18	47.91	0.25	0.16	15.09	20.27
	DW4DO + iDOMap	8.18	21.85	0.37	0.04	6.93	11.00
Scenario 2: Approaching obstacle	DCVM	11.83	30.22	0.39	0.001	14.15	16.75
	DCVM + iDOMap	11.06	28.30	0.39	0.002	0.59	12.18
	DW4DO	–	–	–	–	–	–
	DW4DO + iDOMap	11.84	32.47	0.35	0.081	7.41	10.79
Scenario 3: Lane changing	DCVM	22.549	57.699	0.39	0.001	16.11	19.38
	DCVM + iDOMap	8.43	22.90	0.36	0.084	8.97	15.00
	DW4DO	8.76	22.94	0.37	0.049	9.41	13.54
	DW4DO + iDOMap	8.42	22.787	0.36	0.056	10.70	13.52

**Table 8:** Real experiments: Results.

Experiment	Algorithm	d	t	$\bar{v}$	$\sigma_{v}^{2}$	$\bar{\left|\omega \right|}$	$\sigma_{\omega }^{2}$
Scenario 1: Person overtaking	DCVM	7.61	19.30	0.39	0.043	0.97	2.25
	DCVM + iDOMap	7.63	19.34	0.39	0.045	1.67	4.55
	DW4DO	7.62	19.64	0.38	0.046	2.47	3.92
	DW4DO + iDOMap	7.52	18.96	0.39	0.043	0.71	1.53
Scenario 2: Approaching person	DCVM	6.15	18.52	0.33	0.134	9.06	14.33
	DCVM + iDOMap	6.63	17.40	0.38	0.062	9.75	14.12
	DW4DO	6.18	18.29	0.33	0.127	7.29	8.30
	DW4DO + iDOMap	10.71	28.20	0.37	0.054	10.95	11.90
Scenario 3: Lane changing	DCVM	7.68	21.55	0.35	0.120	4.99	7.98
	DCVM + iDOMap	7.80	21.99	0.34	0.122	5.10	8.56
	DW4DO	7.99	20.96	0.37	0.053	6.81	10.73
	DW4DO + iDOMap	7.96	20.89	0.37	0.057	6.22	9.18

## References

[1] Montemerlo M., Becker J., Bhat S., Dahlkamp H., Dolgov D., Ettinger S., Haehnel D., Hilden T., Hoffmann G., Huhnke B. (2008). Junior: The Stanford Entry in the Urban Challenge. J. Field Robot..

[2] Thrun S., Montemerlo M., Dahlkamp H., Stavens D., Aron A., Diebel J., Fong P., Gale J., Halpenny M., Hoffmann G. (2006). Winning the DARPA Grand Challenge. J. Field Robot..

[3] Schleicher D., Bergasa L.M., Ocaña M., Barea R., López E. (2010). Low-cost GPS sensor improvement using stereovision fusion. Signal Process..

[4] Hentschel M., Wulf O., Wagner B. A GPS and laser-based localization for urban and non-urban outdoor environments. Proceedings of the 2008 IEEE/RSJ International Conference on Intelligent Robots and Systems (IROS 2008).

[5] Thrun S., Fox D., Burgard W. (1998). A probabilistic approach to concurrent mapping and localization for mobile robots. Auton. Robot..

[6] Bresson G., Alsayed Z., Yu L., Glaser S. (2017). Simultaneous Localization and Mapping: A Survey of Current Trends in Autonomous Driving. IEEE Trans. Intell. Veh..

[7] Bekris K.E., Click M., Kavraki E.E. Evaluation of algorithms for bearing-only SLAM. Proceedings of the IEEE International Conference on Robotics and Automation (ICRA’06).

[8] Fox D., Burgard W., Thrun S. (1997). The dynamic window approach to collision avoidance. Robot. Autom. Mag. IEEE.

[9] Durham J.W., Bullo F. Smooth Nearness-Diagram Navigation. Proceedings of the 2008 IEEE/RSJ International Conference on Intelligent Robots and Systems.

[10] López J., Otero C., Sanz R., Paz E., Molinos E., Barea R. A new approach to local navigation for autonomous driving vehicles based on the curvature velocity method. Proceedings of the 2019 International Conference on Robotics and Automation (ICRA).

[11] Elfes A. (1989). Using Occupancy Grids for Mobile Robot Perception and Navigation. Computer.

[12] Chatila R., Laumond J. Position referencing and consistent world modeling for mobile robots. Proceedings of the 1985 IEEE International Conference on Robotics and Automation.

[13] López M., Bergasa L., Barea R., Escudero M. (2005). A Navigation System for Assistant Robots Using Visually Augmented POMDPs. Auton. Robot..

[14] Missura M., Bennewitz M. Predictive Collision Avoidance for the Dynamic Window Approach. Proceedings of the 2019 International Conference on Robotics and Automation (ICRA).

[15] Molinos E.J., Llamazares Á., Ocaña M. (2019). Dynamic window based approaches for avoiding obstacles in moving. Robot. Auton. Syst..

[16] Llamazares Á., Molinos E.J., Ocaña M. (2020). Detection and Tracking of Moving Obstacles (DATMO): A Review. Robotica.

[17] Dempster A.P. (1967). Upper and Lower Probabilities Induced by a Multivalued Mapping. Ann. Math. Statist..

[18] Kurdej M., Moras J., Cherfaoui V., Bonnifait P. (2012). Map-Aided Fusion Using Evidential Grids for Mobile Perception in Urban Environment. Belief Functions: Theory and Applications: Proceedings of the 2nd International Conference on Belief Functions, Compiegne, France, 9–11 May 2012.

[19] Kurdej M., Moras J., Bonnifait P., Cherfaoui V. (2015). Map-Aided Evidential Gridsfor Driving Scene Understanding. IEEE Intell. Transp. Syst. Mag..

[20] Moras J., Cherfaoui V., Bonnifait P. Evidential grids information management in dynamic environments. Proceedings of the 17th International Conference on Information Fusion (FUSION).

[21] Coué C., Pradalier C., Laugier C., Fraichard T., Bessiere P. (2006). Bayesian Occupancy Filtering for Multitarget Tracking: An Automotive Application. Int. J. Robot. Res..

[22] Saval-Calvo M., Medina-Valdés L., Castillo-Secilla J.M., Cuenca-Asensi S., Antonio M.Á., Villagrá J. (2017). A Review of the Bayesian Occupancy Filter. Sensors.

[23] Llamazares A., Ivan V., Molinos E., Ocaña M., Vijayakumar S. (2013). Dynamic Obstacle Avoidance Using Bayesian Occupancy Filter and Approximate Inference. Sensors.

[24] Kaasalainen S., Jaakkola A., Kaasalainen M., Krooks A., Kukko A. (2011). Analysis of Incidence Angle and Distance Effects on Terrestrial Laser Scanner Intensity: Search for Correction Methods. Remote Sens..

[25] Kneip L., Tâche F., Caprari G., Siegwart R. (2009). Characterization of the Compact Hokuyo URG-04LX 2D Laser Range Scanner. Proceedings of the 2009 IEEE International Conference on Robotics and Automation (ICRA’09), Kobe, Japan, 12–17 May 2009.

[26] Kawata H., Miyachi K., Hara Y., Ohya A., Yuta S. A method for estimation of lightness of objects with intensity data from SOKUIKI sensor. Proceedings of the IEEE International Conference on Multisensor Fusion and Integration for Intelligent Systems (MFI 2008).

[27] García J., Gardel A., Bravo I., Lázaro J.L., Martínez M. (2013). Tracking People Motion Based on Extended Condensation Algorithm. IEEE Trans. Syst. Man Cybern. Syst..

[28] Li B., Yang C., Zhang Q., Xu G. Condensation-based multi-person detection and tracking with HOG and LBP. Proceedings of the 2014 IEEE International Conference on Information and Automation (ICIA).

[29] Doucet A., Godsill S., Andrieu C. (2000). On sequential Monte Carlo sampling methods for Bayesian filtering. Stat. Comput..

[30] Isard M., Blake A. (1998). Icondensation: Unifying low-level and high-level tracking in a stochastic framework. Computer Vision—ECCV’98: 5th European Conference on Computer Vision, Freiburg, Germany, 2–6 June 1998 Proceedings, Volume I.

[31] Ripley B.D. (1987). Stochastic Simulation.

[32] Isard M.A. (1998). Visual Motion Analysis by Probabilistic Propagation of Conditional Density. Ph.D. Thesis.

[33] Mekhnacha K., Mao Y., Raulo D., Laugier C. Bayesian occupancy filter based ”Fast Clustering-Tracking” algorithm. Proceedings of the IEEE/RSJ 2008 International Conference on Intelligent Robots and Systems.

[34] Wang C.C. (2004). Simultaneous Localization, Mapping and Moving Object Tracking. Ph.D. Thesis.

[35] Nègre A., Rummelhard L., Laugier C. Hybrid sampling Bayesian Occupancy Filter. Proceedings of the 2014 IEEE Intelligent Vehicles Symposium Proceedings.

[36] Mertz C., Navarro-Serment L.E., Duggins D., Gowdy J., MacLachlan R., Rybski P., Steinfeld A., Suppe A., Urmson C., Vandapel N. (2013). Moving object detection with laser scanners. J. Field Robot..

[37] Molinos E., Llamazares Á., Ocaña M., Herranz F. Dynamic Obstacle Avoidance based on Curvature Arcs. Proceedings of the 2014 IEEE/SICE International Symposium on System Integration.

[38] Hornung A., Wurm K.M., Bennewitz M., Stachniss C., Burgard W. (2013). OctoMap: An Efficient Probabilistic 3D Mapping Framework Based on Octrees. Auton. Robot..

[39] Tatoglu A., Pochiraju K. Point cloud segmentation with LIDAR reflection intensity behavior. Proceedings of the 2012 IEEE International Conference on Robotics and Automation.

[40] Cáceres Hernández D., Hoang V., Jo K. Lane Surface Identification Based on Reflectance using Laser Range Finder. Proceedings of the 2014 IEEE/SICE International Symposium on System Integration.

[41] Caltagirone L., Scheidegger S., Svensson L., Wahde M. Fast LIDAR-based road detection using fully convolutional neural networks. Proceedings of the 2017 IEEE Intelligent Vehicles Symposium (IV).

